# Supplementary material for: The genome sequence of a scirtid beetle, Prionocyphon serricornis (Muller, 1821) (Coleoptera: Scirtidae)
Source: Wellcome Open Res. 2026 Sep 24;11:747. [Version 1] doi: 10.12688/wellcomeopenres.27928.1 (PMC13731894; doi:10.12688/wellcomeopenres.27928.1)
Supplement: Peer review history

# Reviewer report: Reviewer response for version 1

**DOI:** 10.21956/wellcomeopenres.30728.r173418

**Published:** 2026-11-03

**Authors:** Renee Ali

**Affiliations:** 1 Johns Hopkins University Bloomberg School of Public Health, Baltimore, Maryland, USA

The high-quality chromosome-level genome assembly presented in the study is an important genetic resource for *Prionocyphon serricornis*.  The sequencing and assembly methods are clearly described, with the main limitation being the incomplete resolution of some chromosome structures. However, this genome assembly provides a useful foundation for future comparative and evolutionary studies.

Are the datasets clearly presented in a usable and accessible format, and the assembly and annotation available in an appropriate subject-specific repository?

Yes

Are sufficient details of the sequencing and extraction, software used, and materials provided to allow replication by others?

Yes

Are the rationale for sequencing the genome and the species significance clearly described?

Yes

Are the protocols appropriate and is the work technically sound?

Yes

Reviewer Expertise:

mitocondrial genome assembly, applications, phylogeny

I confirm that I have read this submission and believe that I have an appropriate level of expertise to confirm that it is of an acceptable scientific standard.

# Reviewer report: Reviewer response for version 1

**Published:** 2026-09-24

**Authors:** Automated Benchmarking

**Affiliations:** 1 Wellcome Sanger Institute, Hinxton, UK

As this article is a Genome Note, Automated Benchmarking has been used to determine whether the reported assembly passes four defined checks established by the Earth BioGenome Project: the raw and assembled data are publicly available, the Earth BioGenome Project three-part metric is met, the k-mer completeness exceeds 90%, and the BUSCO score passes the stated threshold. For Prionocyphon serricornis (icPriSerr1), all four checks passed. Data available: pass. ENA study PRJEB80659, raw-read accessions ERR13762700 and ERR13732206, and assembly accessions GCA_965648515.1 and GCA_965648555.1 are public. Earth BioGenome Project three-part metric: pass. Haplotype 1 is reported as 6.C.Q55, meeting the 6.C.Q40 reference standard. k-mer completeness: pass. The combined haplotype assemblies have 97.43% k-mer completeness, exceeding the Earth BioGenome Project target of 90%. BUSCO: pass. Haplotype 1, which is proposed as the reference assembly for this species, has 98.8% complete BUSCOs (97.8% single-copy and 1.0% duplicated). BUSCO software version: 5.8.3; lineage dataset: endopterygota_odb10. It meets the target of greater than 90% complete and single-copy BUSCOs. This decision uses values reported in the Genome Note and public accession records.

Reviewer Expertise:

NA

I confirm that I have read this submission and believe that I have an appropriate level of expertise to confirm that it is of an acceptable scientific standard.
